# Amelioration of Saline Alkali–Soil and Improvement of Soybean Performance by Organically Modified Coal Gangue

**DOI:** 10.3390/nano16211436

**Published:** 2026-11-06

**Authors:** Qi Zhao, Zhipeng Yao, Yusheng Niu, Xiangjie Guo, Kunpeng Cui

**Affiliations:** 1 Institute of Biomedical Engineering, College of Life Sciences, Qingdao University, Qingdao 266071, China; 2 Binzhou Academy of Agricultural Sciences, Binzhou 256600, China; 3 Research Institute of Modern Agricultural Industry Innovation in Yellow River Delta Saline-Alkali Land, Dongying Vocational College, Dongying 257000, China; 4 Shandong Minsheng Group Co., Ltd., Jinan 250000, China

**Keywords:** modified coal gangue, saline–alkali soil, soil properties, soybean growth, soil remediation

## Abstract

Growing global concern over soil salinization has drawn considerable attention to the remediation and amelioration of saline–alkali soils. Coal gangue (M) can be applied to improve saline–alkali soils; nevertheless, its amelioration performance is subject to certain limitations. Organically modified coal gangue (GM) was prepared using alcohol ethoxylate-7 (AEO-7) as the primary modifier, and both M and GM were mixed with a saline–alkali soil at 10%, 20%, 30% and 40% (*w*/*w*) in a 28-day incubation and a 28-day soybean pot experiment. At 28 d, compared with the CK group, the GM40% treatment significantly increased the available potassium and alkali-hydrolyzable nitrogen in the saline–alkali soil by 49.91% and 97.10%, respectively, and increased soil organic matter by 10% compared with M40%. The GM groups decreased soil pH relative to CK, alleviated M-induced available phosphorus deficiency, and increased soil urease, peroxidase and catalase activities by 467.77%, 140.37% and 76.29%, respectively. Plant height, shoot dry weight and root dry weight increased by 23.24%, 102.22% and 208.05%. Amplicon sequencing showed that the modified material increased bacterial richness and shifted bacterial community composition. Thus, the organically modified gangue improved soil nutrient pools and soybean performance relative to raw gangue.

## 1. Introduction

Soil salinization is a major problem in land management, especially in arid, semi-arid, and coastal areas [1]. These soils create complex, multi-level challenges for farming and ecosystems [2]. High salt levels cause physiological drought and osmotic stress in plants. Excess specific ions can be toxic and upset nutrient balance [3]. Exchangeable sodium breaks down soil structure by destroying aggregates, which reduces permeability and hinders seed germination and root growth [4]. With poor texture, bad drainage, and low nutrients, these soils are prone to both drought and waterlogging. As a result, crop yields drop, land degrades [5], biodiversity is lost [6], and desertification speeds up [7].

General strategies for ameliorating saline–alkali land include planting salt-tolerant species, soil capping and applying amendments [8]. Phytoremediation is typically slow [9], and soil capping, while rapid in effect, involves high costs. In comparison, applying soil amendments presents a cost-effective and efficient alternative [10]. Coal gangue, a major byproduct of coal mining [11], is currently processed into building materials [12], used for thermal power generation [13], or backfilled into mining subsidence areas [14]. However, these approaches consume less than 30% of the annual accumulation [15]. Previous work suggests that coal gangue can improve soil bulk density and porosity in saline–alkali soils while also enhancing microbial abundance and enzyme activities [16]. Given its large supply and low cost, coal gangue holds promise as a soil amendment for saline–alkali land. However, raw coal gangue has inconsistent remediation effects that depend heavily on particle size and application rate. It may reduce soil aeration, suppress microbial activity, and inhibit plant growth [17]. To enhance the resource utilization of coal gangue, modified coal gangue has been developed. Traditional modification strategies mainly include calcination [18], acid treatment [19] and alkaline activation. However, these methods are primarily oriented toward industrial fields (e.g., producing cementitious materials or extracting metals [20]) and generally suffer from high energy consumption [21] and severe pollution. These routes act by destroying or reconstructing mineral phases to release or immobilize nutrients, and their performance as saline–alkali soil amendments has not yet been evaluated. By contrast, interfacial routes that alter particle wettability and the gangue–solution interaction at low modifier loading have received comparatively little attention.

Alcohol ethoxylate-7 (AEO-7) belongs to nonionic surfactants, consisting of hydrophobic long-chain fatty alcohol segments and hydrophilic polyoxyethylene chains in its molecular architecture [22]. This amphipathic structure endows it with outstanding surface activity, making it widely used as a modifier to reduce interfacial tension [23] and improve dispersion [24]. To achieve non-destructive interfacial regulation in this study, AEO-7 was selected as the modifier. AEO-7 maintains surface activity under the high pH and ionic strength of saline–alkali soils. Furthermore, its amphiphilic structure enables a dual anchoring mechanism: the hydrophilic EO chains form hydrogen bonds with the gangue’s surface silanol/aluminol groups, while the hydrophobic tail interacts with the carbonaceous surfaces. This mild, aqueous modification route is expected to alter the wettability of coal gangue, facilitating water infiltration and nutrient transport when applied to saline–alkali soils. Therefore, we further hypothesized that this modified surface would strengthen gangue–soil interactions and improve soil physicochemical properties, enzyme activities, microbial community structure, and soybean performance.

## 2. Materials and Methods

### 2.1. Modification of Coal Gangue

The coal gangue (M) used in this experiment was obtained from the Datong Coal Mine in Shanxi Province. Prior to use, it was ground and passed through an 80-mesh sieve (<0.18 mm). For modification, 10 g of AEO-7 (Shanghai Aladdin Biochemical Technology Co., Ltd., Shanghai, China) was first mixed with 800 g of deionized water to form micelles. Subsequently, 1 g of Na_2_MoO_4_ (Shanghai Macklin Biochemical Co., Ltd., Shanghai, China) was added and dissolved under stirring, followed by the addition of 15 g of MgSO_4_ (Shanghai Macklin Biochemical Co., Ltd., Shanghai, China). After the reagents were fully mixed, 1000 g of coal gangue powder was added to the solution. After standing for 12 h, the precipitated coal gangue was filtered and dried at a low temperature of 40 °C for 48 h to obtain the modified coal gangue (GM).

Major elemental contents of M samples were determined via X-ray fluorescence (XRF) (ARL PERFORM’X, Thermo Fisher Scientific, Waltham, MA, USA). Scanning electron microscopy (SEM) (JSM-7800F, JEOL Ltd., Tokyo, Japan) captured micro-morphological features of specimen surfaces. Fourier-transform infrared spectroscopy (FTIR) (Nicolet iS50, Thermo Fisher Scientific, Waltham, MA, USA) identified surface functional groups and variations in chemical bonds. Contact angle measurements distinguished surface hydrophilicity and hydrophobicity between raw and modified coal gangue. Low-temperature nitrogen adsorption (BET) (Autosorb iQ3, Quantachrome Instruments, Boynton Beach, FL, USA) was used to quantify specific surface area, total pore volume and pore size distribution.

### 2.2. Experimental Design

The soybean cultivar used in this study was Williams 82, obtained from Jifeng Biological Company. The saline–alkali soil was collected from Hekou District, Dongying City (37.88° N, 118.52° E). Surface soil samples were collected at a 20 cm depth. During the study period and immediately prior to sampling, the climate in the sampling area was stable, and no natural disasters occurred. The soil was air-dried and passed through a 2 mm sieve before use. Initial soil properties: pH, 8.25; alkali-hydrolyzable nitrogen, 18.20 mg·kg^−1^; available phosphorus, 13.21 mg·kg^−1^; available potassium, 102.30 mg·kg^−1^; and moisture content of approximately 8%. To evaluate the effect of GM on soil amelioration and soybean growth, we conducted a pot experiment in a greenhouse with three replicates per treatment. The greenhouse conditions were maintained at a temperature of 22.8 °C, with a photoperiod of 14 h of light and 10 h of darkness and a relative humidity ranging from 50% to 60%.

Soybean seeds were dried in a constant-temperature incubator (HS-150, Huaxixinrui (Qingdao) Analytical Instrument Co., Ltd., Qingdao, China) at 37 °C for 2~3 d. Prior to planting, the seeds were surface-sterilized by immersion in a 1% NaClO (Shanghai Macklin Biochemical Co., Ltd., Shanghai, China) solution for 15 min, followed by thorough rinsing with deionized water [25]. Germination was conducted in seedling trays using sterilized substrate soil, with seeds planted at a depth of approximately 2 cm.

The experimental pots measured 10 cm in diameter and 9 cm in height. The growth substrate consisted of a mixture of sieved saline–alkali soil and coal gangue in various ratios. Each pot contained 500 g of the homogenized substrate mixture, which was allowed to equilibrate for 3 days before use. Coal gangue was divided into two groups according to its type: coal gangue (M group) or modified coal gangue (GM group). For each type, four application rates (10%, 20%, 30%, and 40%) were tested, resulting in the following treatment groups: (1) M10%, M20%, M30%, and M40% and (2) GM10%, GM20%, GM30%, and GM40%. A total of nine treatment groups were established, including a control group (CK group) consisting solely of saline–alkali soil. Each treatment was replicated six times, with three replicates used for soybean cultivation and the remaining three for determining soil physicochemical properties.

Soybean seedlings were transplanted into experimental pots upon full expansion of the second true leaf. Two seedlings with consistent growth status, plant size and developmental stage were retained in each pot. Appropriate watering was supplied after transplanting to maintain soil water content at approximately 70% of field capacity. Plant morphological and physiological–biochemical parameters of soybean were determined 28 d after transplantation.

After the soil was amended with coal gangue and subjected to a 3 d pre-incubation, soil sampling was conducted on days 0, 7, 14, 21 and 28. At each sampling time point, mixed soil samples (25 g) were collected via the five-point sampling method. Subsequently, 15 g of each fresh soil sample was air-dried under natural ambient conditions, followed by grinding and sieving pretreatment for subsequent determination of soil physicochemical properties and enzyme activities. The remaining 10 g of fresh soil was stored at −80 °C for subsequent analysis of the soil microbial community. Soil alkali-hydrolyzable nitrogen content was determined using the diffusion method [26]. Available phosphorus was extracted with 0.5 mol/L NaHCO_3_ (Shanghai Macklin Biochemical Co., Ltd., Shanghai, China) at pH 8.5 (Olsen method) and determined colorimetrically by the molybdenum blue method. Available potassium was extracted with 1 mol L^−1^ ammonium acetate (pH 7.0) (Shanghai Macklin Biochemical Co., Ltd., Shanghai, China) and determined by flame photometry. Soil organic matter was assayed using the potassium dichromate external heating method. Soil pH was determined in a soil–water suspension at a mass-to-volume ratio of 1:5.

### 2.3. Determination of Soybean Growth and Development Parameters

At the end of the experimental period, soybean plant samples were harvested from each treatment. Three uniformly growing soybean plants were randomly selected per treatment group for the determination of plant growth indicators, photosynthetic properties, chlorophyll concentration and nitrogen content. Plant height and root length were manually measured, while plant fresh and dry weights were quantified via the gravimetric method. For dry weight measurement, sampled plants were subjected to an initial high-temperature deactivation at 100 °C for 15 min, followed by continuous drying at 37 °C for 72 h until a constant mass was obtained. The harvested fresh seedlings were dissected into roots, stems and leaves. The separated plant tissues were wrapped with aluminum foil, immediately snap-frozen in liquid nitrogen, and preserved in an ultra-low-temperature freezer (Forma 900, Thermo Fisher Scientific, Waltham, MA, USA) at −80 °C for subsequent biochemical analyses. A chlorophyll meter (SPAD-502Plus, Konica Minolta, Tokyo, Japan) was utilized to record leaf SPAD readings and determine plant nitrogen concentration. Superoxide dismutase (SOD) activity was determined via the nitroblue tetrazolium photoreduction method. Catalase (CAT) and peroxidase (POD) activities were quantified using assay kits from Solarbio (Beijing Solarbio Science & Technology Co., Ltd., Beijing, China).

Soil samples were cryopreserved after collection and shipped to Novogene (Novogene Co., Ltd., Beijing, China) for 16S rRNA high-throughput amplicon sequencing. Target fragments were amplified to construct libraries, which were subjected to paired-end sequencing on the Illumina NovaSeq platform. Raw reads were assembled and filtered, followed by ASV denoising for taxonomic annotation and abundance profiling. Alpha and beta diversity analyses were performed to decipher soil microbial community composition and intergroup structural discrepancies.

### 2.4. Data Processing and Statistical Analysis

Normality and homogeneity of variance were assessed using the Shapiro–Wilk and Levene tests prior to statistical analysis. Variables meeting these assumptions were analyzed using two-way analysis of variance to evaluate the effects of amendment treatment, time, and their interaction on soil physicochemical properties and enzyme activities, followed by Tukey’s post hoc test. For soybean physiological and growth parameters, one-way analysis of variance followed by Tukey’s post hoc test was employed to analyze the effects of different amendment treatments. Statistical significance was set at *p* < 0.05. Results are reported as mean ± standard deviation (SD), with three replicates per treatment (*n* = 3). Data analysis was conducted using SPSS 18. Graphs were assembled using GraphPad Prism 10 and compiled using Adobe Illustrator 2024.

## 3. Results

### 3.1. Physicochemical Properties of Modified Coal Gangue

The mass percentages of major elements in M and GM are presented below (Table 1). Relative to M, the C and S contents in the GM showed relative increases of 4.51% and 17.89%, respectively. The Si and Al contents showed relative decreases of 20.16% and 18.55%, respectively. The Fe content exhibited a negligible relative decrease of 0.24%, indicating negligible difference between the two groups.

The mass percentages of the corresponding oxides are further presented in Table 2. Compared with M, GM exhibited a 4.51% relative increase in CO_2_ and a 17.89% relative increase in SO_3_. SiO_2_ and Al_2_O_3_ decreased by 20.16% and 18.55% (relative), respectively. Fe_2_O_3_ only declined by 0.25%, with negligible variation between the two groups.

The trace element contents of the two types of coal gangue are shown in Table 3. The pH of the coal gangue used in this study is 8.76. At this pH, the contents of As, Pb, Cd, Cr, Cu, and Ni in both coal gangue samples are lower than the risk screening values for soil contamination of agricultural land. The elemental contents comply with the agricultural land requirements of the Soil Environmental Quality—Risk Control Standard for Soil Contamination of Agricultural Land (Trial). These materials pose a low risk to the quality and safety of agricultural products, crop growth, and the soil ecological environment. They cause essentially no environmental pollution and can be used for soil improvement.

**Figure 1:**
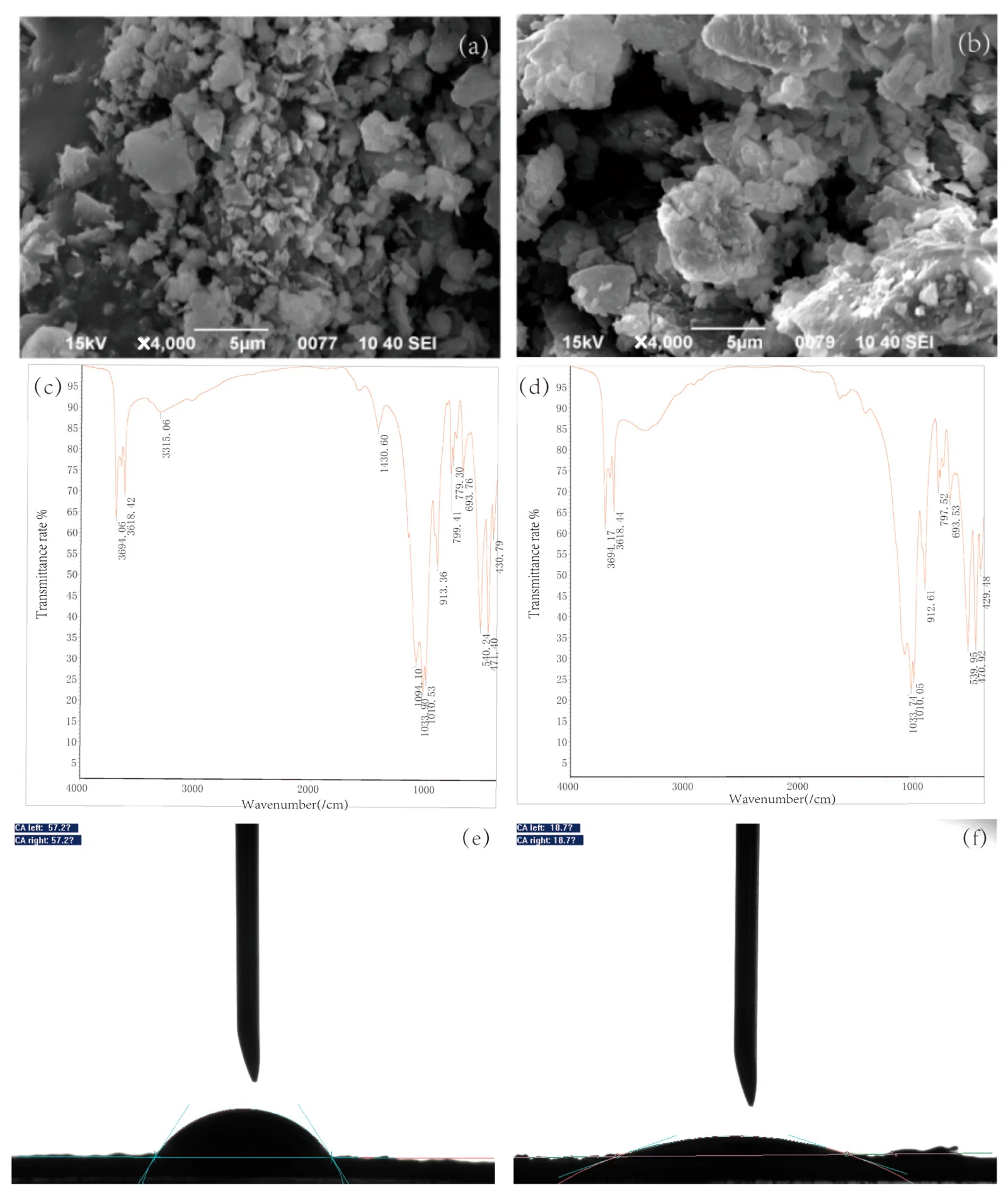
SEM images of M (**a**) and GM (**b**); FTIR spectra of M (**c**) and M (**d**); and contact-angle images of M (**e**) and GM (**f**).

SEM images revealed that the unmodified M particles exhibited an irregular and isolated morphology, with a particle size distribution predominantly below 5 µm (Figure 1a). After modification, the GM particles showed noticeable agglomeration, resulting in a substantial increase in overall particle size (Figure 1b).

The FTIR spectra of the M group (Figure 1c) and organically modified GM (Figure 1d) were recorded over the wavenumber range of 4000–400 cm^−1^. Compared with the M group, GM exhibited additional absorption bands in the 2800–3000 cm^−1^ region, corresponding to alkyl C-H stretching vibrations. The characteristic absorption bands of coal gangue at 1000–1100, 700–950, and 400–600 cm^−1^ were largely retained in GM, with only minor shifts.

The contact-angle results are presented above, where Figure 1e represents M and Figure 1f represents GM. The M sample exhibits a contact angle of approximately 57.2°, indicating hydrophilic properties. In contrast, the GM sample shows a significantly lower contact angle of about 16.4°, demonstrating strong hydrophilicity.

The pore-structure parameters of the two coal gangue samples are compared in Table 4. The specific surface area of GM decreased from 2.2674 m^2^/g to 1.6626 m^2^/g after modification, representing a reduction of approximately 26.73%, which is consistent with the SEM results. Compared to M, GM exhibited an increase in pore volume from 0.0065 cm^3^/g to 0.0077 cm^3^/g, corresponding to an increase of about 18.55%, and an increase in average pore diameter from 11.4669 nm to 18.5252 nm, corresponding to an increase of approximately 61.62%.

### 3.2. Effects of Different Coal Gangue Application Rates on the Physicochemical Properties of Saline–Alkali Soil

**Figure 2:**
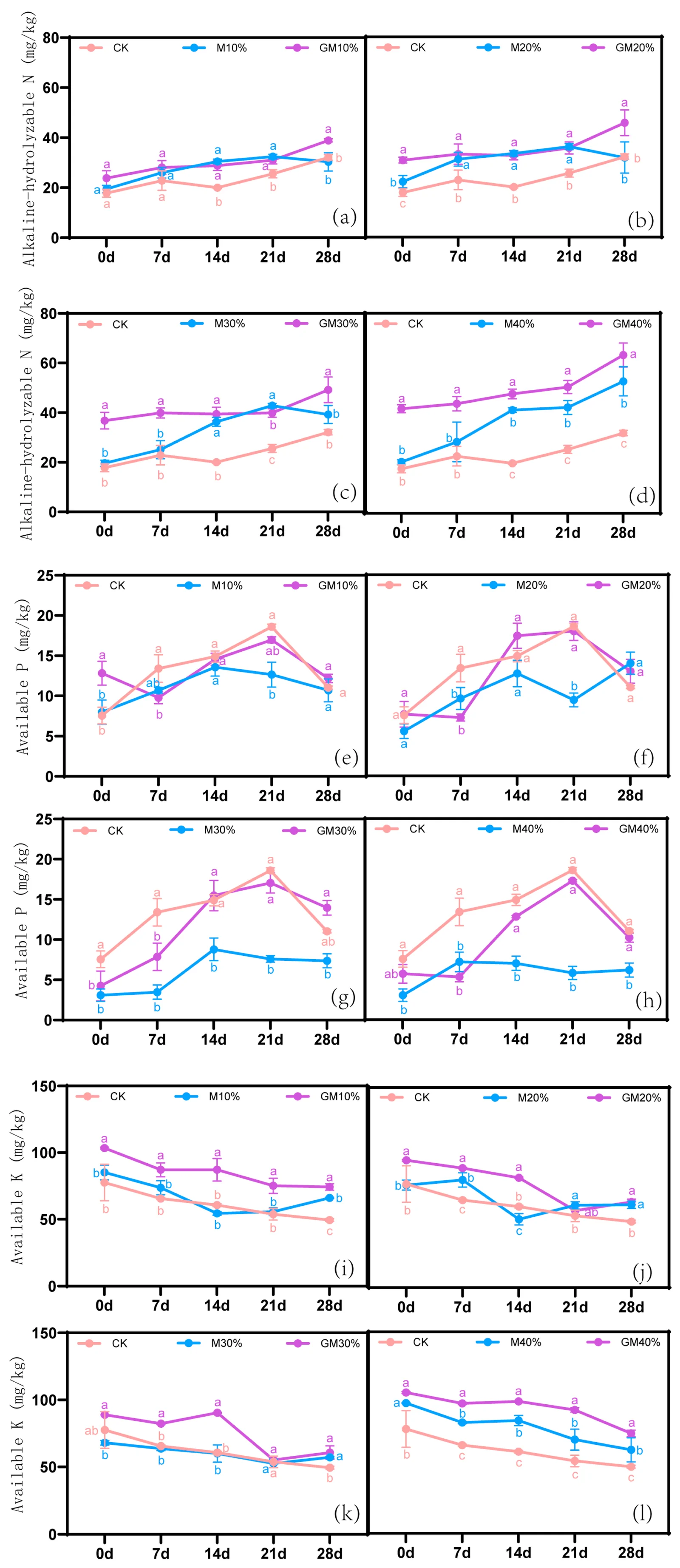
Temporal dynamics of AHN (**a**–**d**), available phosphorus (**e**–**h**), and available potassium (**i**–**l**) contents in saline–alkali soil as affected by different application rates of coal gangue materials. **CK:** unamended saline–alkali soil (control); **M:** saline–alkali soil amended with unmodified coal gangue; **GM:** saline–alkali soil amended with organically modified coal gangue. For each parameter, the bars from left to right represent application rates of 10%, 20%, 30%, and 40%, respectively. The lines and error bars show means ± SD (*n* = 3). Different lowercase letters indicate significant differences under different treatments (*p* < 0.05).

Coal gangue addition altered several soil chemical properties over the 28 d incubation (Figure 2). Both M and GM increased alkali-hydrolyzable nitrogen (AHN) relative to CK, and the increase generally became more pronounced with increasing application rate and incubation time. By day 28, the M group increased AHN by up to 64.68%, whereas the GM group increased it by 19.37–96.87%, with GM40% showing the highest value. In contrast, available phosphorus decreased under both materials, but the decline was smaller under the GM group (7.27–10.91%) than under the M group (9.01–45.45%). Available potassium also increased with amendment rate; at 40% on day 28, concentrations were 49.49, 62.06, and 74.26 mg/kg in the CK, M, and GM groups, respectively.

**Figure 3:**
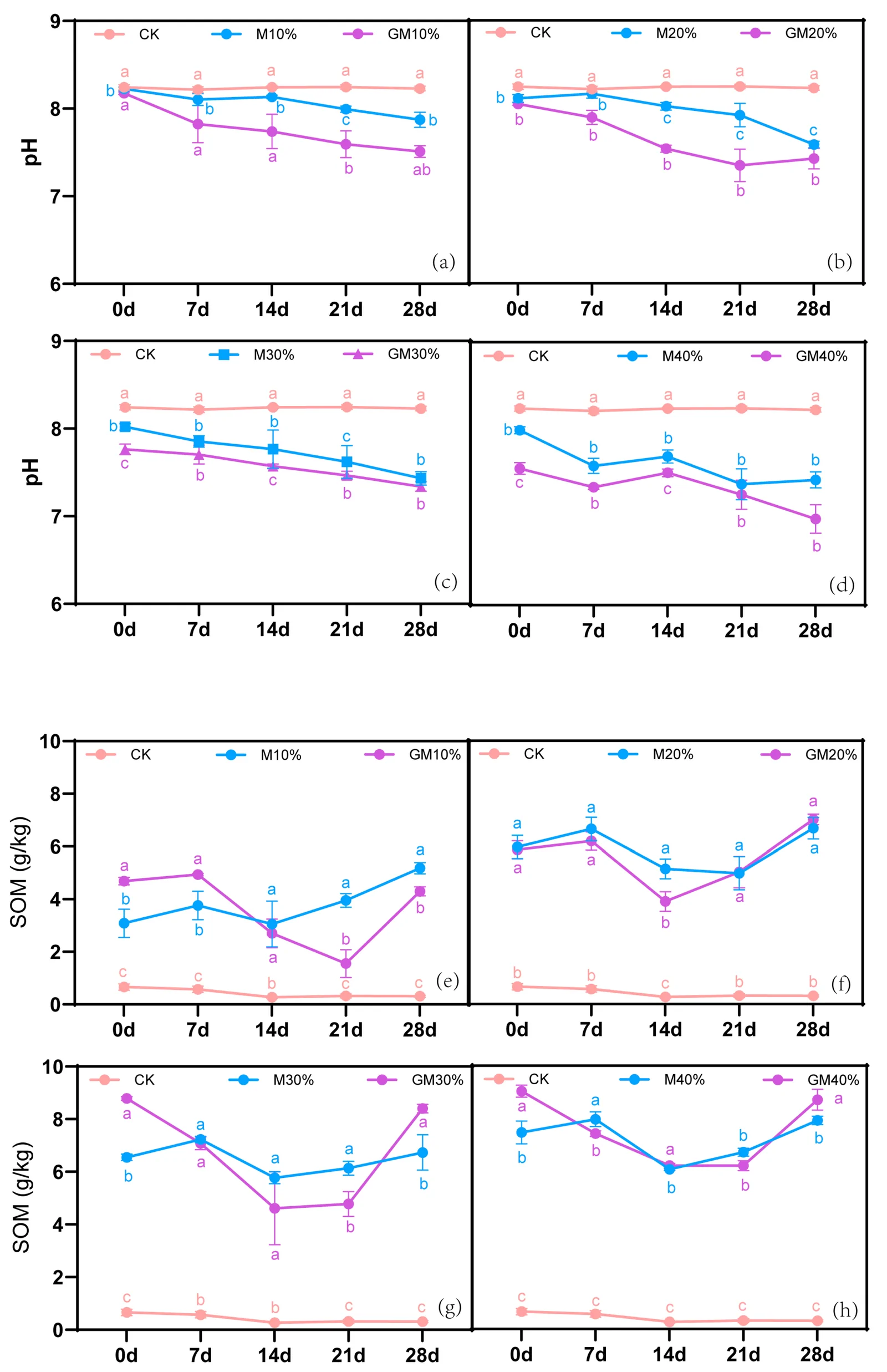
Effects of different material application rates on the temporal variation of pH (**a**–**d**) and SOM (**e**–**h**) content in saline–alkali soil. For each parameter, the bars from left to right represent application rates of 10%, 20%, 30%, and 40%. The lines and error bars show means ± SD (*n* = 3). Different lowercase letters show significant differences among treatments (*p* < 0.05).

Soil pH was reduced by both amendment types relative to CK, with decreases of 8.41–9.39% under the M group and 3.41–14.63% under the GM group (Figure 3a–d). Soil organic matter (SOM) decreased during the first 14 d and increased thereafter. Across the incubation period, SOM was higher in amended soils than in CK, with the largest increase observed under GM40% (Figure 3e–h).

### 3.3. Effects of Varying Coal Gangue Application Rates on Enzyme Activities in Saline–Alkali Soil

**Figure 4:**
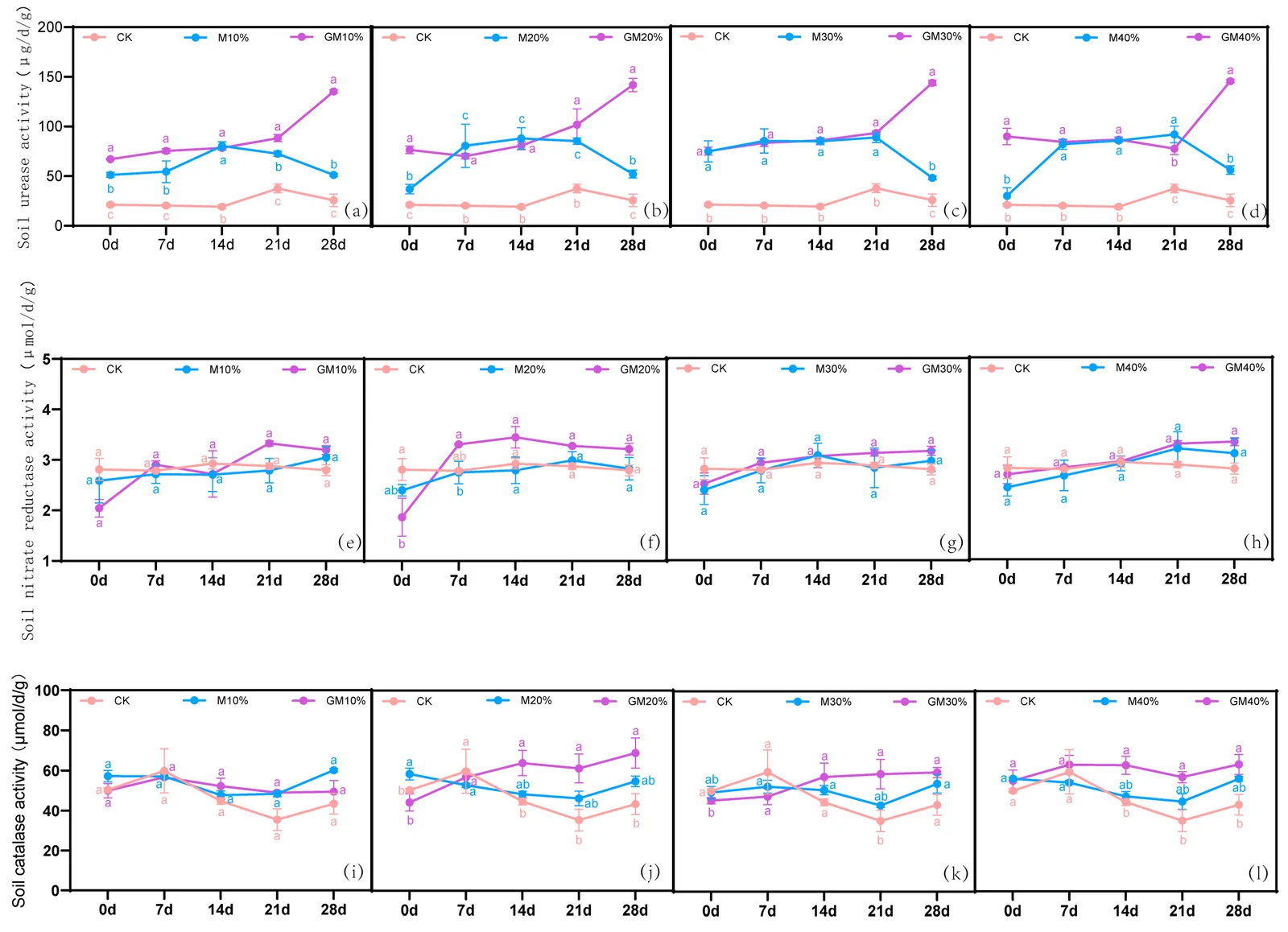
Effects of different material application rates on the activities of soil urease (**a**–**d**), soil nitrate reductase (**e**–**h**), and soil catalase (**i**–**l**) in saline–alkali soil over time. For each parameter, the bars from left to right represent application rates of 10%, 20%, 30%, and 40%. The lines and error bars show means ± SD (*n* = 3). Different lowercase letters indicate significant differences among treatments (*p* < 0.05).

Soil enzyme activities differed among treatments throughout the incubation period (Figure 4). Urease activity was higher under both the M group and GM group than under CK. By day 28, the M group increased urease activity by 99.92–119.21% relative to CK, whereas the GM group increased it by 426.89–467.77%. Nitrate reductase activity in the CK group showed an initial increase followed by a decline, whereas both amendment groups showed an initial increase followed by a more stable phase. After day 7, nitrate reductase activity increased by 16.48–21.98% under the GM group and by 1.10–9.90% under the M group relative to CK. Catalase activity was also higher under the amendment treatments, with GM40% reaching 66.67 μmol/d/g.

### 3.4. Effects of Different Coal Gangue Application Rates on Soybean Physiological and Growth Parameters in Saline–Alkali Soil

**Figure 5:**
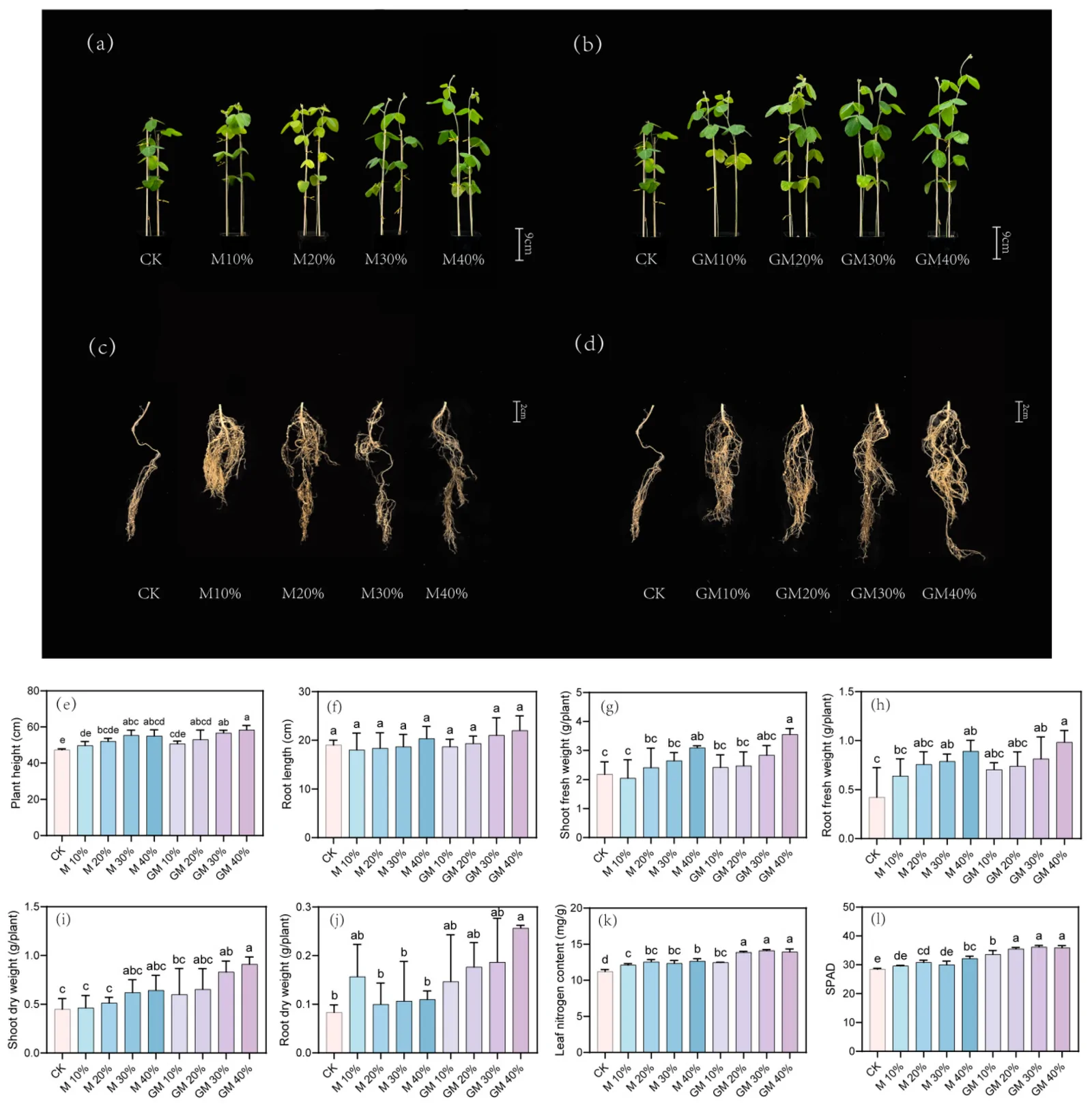
Soybean growth performance under different treatments: (**a**) CK, M10%, M20%, M30%, and M40%; (**b**) CK, GM10%, GM20%, GM30%, and GM40%. Soybean root growth under different treatments: (**c**) CK, M10%, M20%, M30%, and M40%; (**d**) CK, GM10%, GM20%, GM30%, and GM40%. Quantitative parameters of soybean growth: (**e**) plant height, (**f**) root length, (**g**) aboveground fresh weight, (**h**) root fresh weight, (**i**) aboveground dry weight, (**j**) root dry weight, (**k**) leaf nitrogen content, and (**l**) leaf SPAD value across different experimental groups. The bars and error bars show means ± SD (*n* = 3). Different lowercase letters show significant differences among treatments (*p* < 0.05).

A pot experiment was conducted to evaluate the effects of raw gauge and the GM group at different application rates on soybean growth and development in saline–alkali soil. During the 28-day cultivation period, both coal gangue and the GM group applied at rates of 10%, 20%, 30%, and 40% significantly affected soybean growth. Compared with the control and raw coal gangue treatments, the GM group consistently promoted aboveground growth, as reflected by increases in plant height, shoot fresh weight, and shoot dry weight. Among the treatments, GM40% produced the greatest increases in these growth parameters, with plant height, shoot fresh weight, and shoot dry weight increasing by 23.24%, 62.62%, and 102.22% (Figure 5), respectively, relative to the control. Root growth showed a similar response pattern across the application rates, with the greatest root length observed under GM40%. Compared with the control, GM40% increased root fresh weight and root dry weight by 132.29% and 208.05%, respectively (Figure 5).

As illustrated in Figure 5l, SPAD values in both the M group and the GM group increased with higher coal gangue application rates, with the maximum SPAD value observed in GM30%. Compared with the control, the SPAD values increased by 27.01% and 26.03% under the GM30% and GM40% treatments, respectively, with no significant difference between the two treatments.

**Figure 6:**
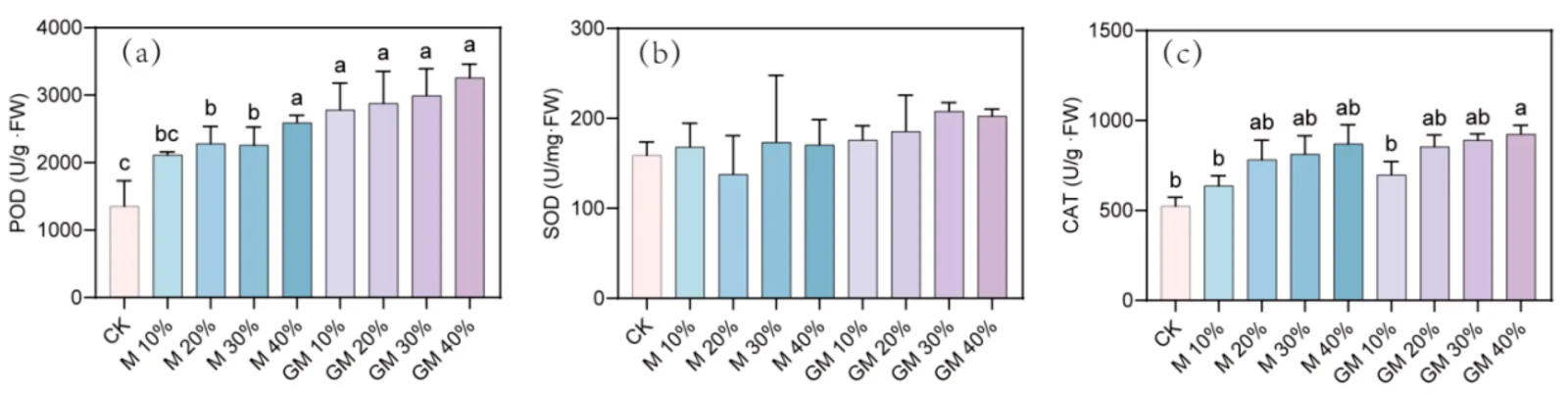
Enzyme activities of soybean in different experimental groups, including peroxidase (**a**), superoxide dismutase (**b**), and catalase (**c**) activities. Bars and error bars show means ± SD (*n* = 3). Different lowercase letters show significant differences among treatments (*p* < 0.05).

Soybean antioxidant enzyme activities responded differently to the GM group treatments (Figure 6). POD activity reached its highest value under GM40%, increasing by 140.37% compared with the control (Figure 6a). SOD activity was also highest under GM30%, reaching 208.20 U/mg, compared with 159.26 U/mg in the control, and was generally higher under GM group treatments than under the corresponding M group (Figure 6b). CAT activity increased progressively with increasing application rate and reached 924.30 U/g under GM40%, representing a 76.29% increase relative to the control (Figure 6c).

**Figure 7:**
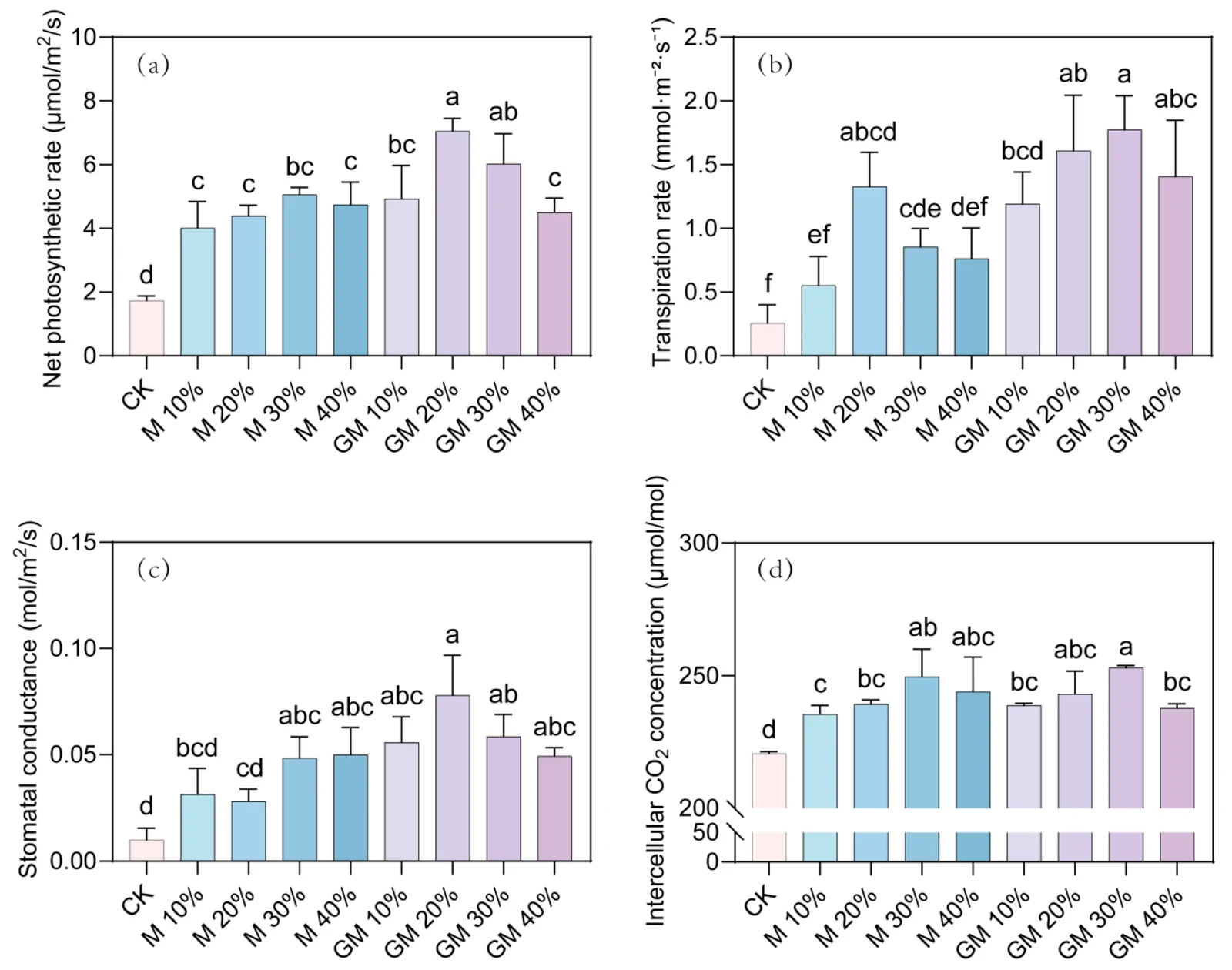
Photosynthetic characteristics of soybean in different experimental groups, including photosynthetic rate (**a**), transpiration rate (**b**), stomatal conductance (**c**), and intercellular CO_2_ concentration (**d**). Bars and error bars represent means ± SD (*n* = 3). Different lowercase letters show significant differences among treatments (*p* < 0.05).

Photosynthetic traits showed clear but non-monotonic responses to the application rate of the GM group (Figure 7). The net photosynthetic rate (Pn) initially increased, then declined with increasing GM application rate, reaching a maximum of 7.03 μmol/m^2^/s under GM20%, which was 313.53% higher than that in CK (Figure 7a). Compared with the corresponding M group, the GM group generally resulted in a higher Pn, stomatal conductance (Gs), and transpiration rate (Tr), with the differences being particularly evident at the 10–30% application rates (Figure 7b–d). Gs and Tr were consistently higher under the GM group than under the corresponding M group treatments, whereas Ci showed a less pronounced difference between the two amendment types. Among the GM group treatments, GM20% produced the highest Pn and Gs values.

### 3.5. Effects of Various Coal Gangue Types and Application Rates on Soil Microbial Communities

**Figure 8:**
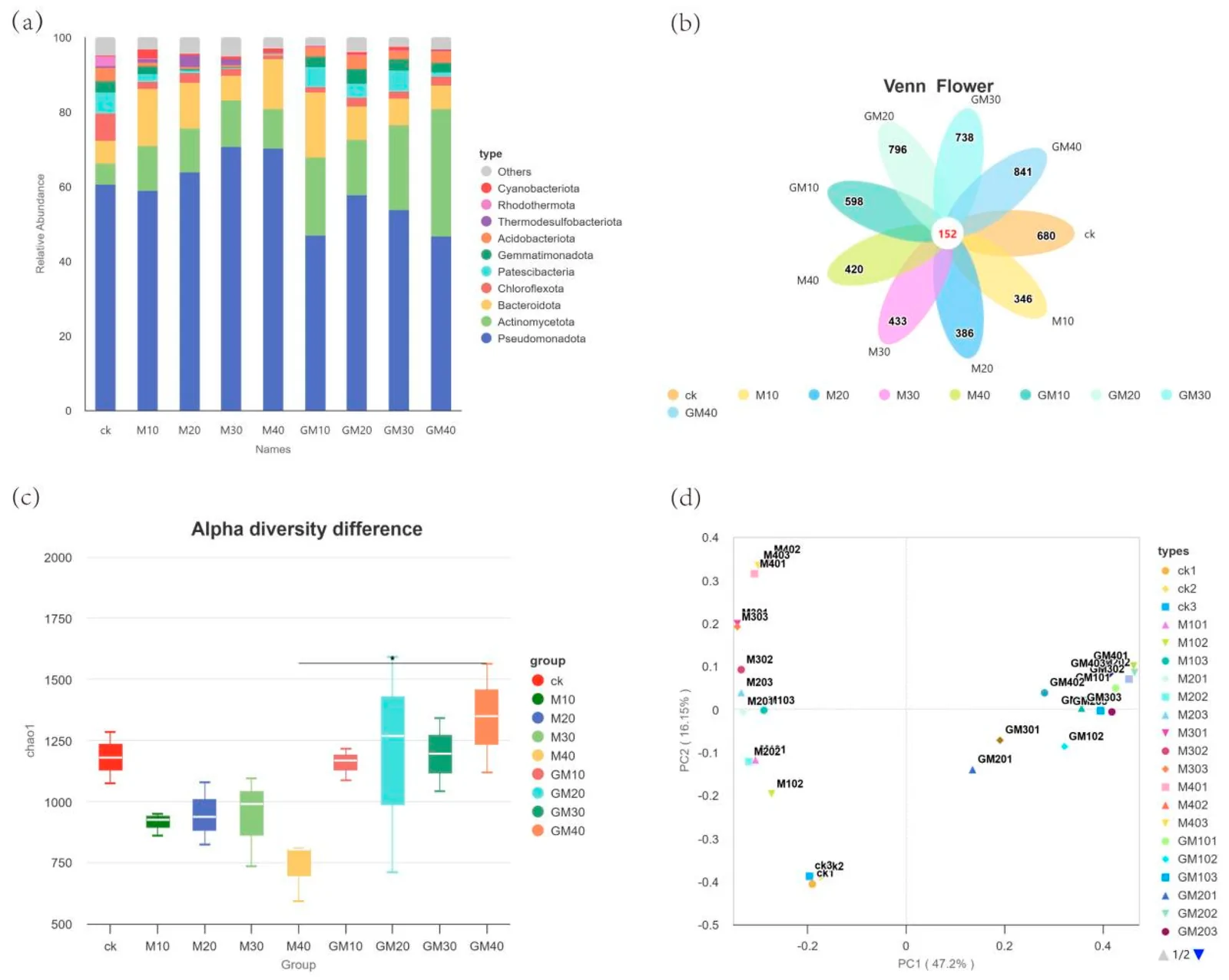
Results of 16S rRNA gene sequencing analysis for each experimental group. (**a**) Relative abundance of bacterial communities at the phylum level under different treatments. (**b**) Venn diagram illustrating shared and unique ASVs among treatments. (**c**) Chao1 richness index of soil bacterial communities under different treatments. (**d**) Principal coordinate analysis (PCoA) of soil bacterial community structure based on Bray–Curtis dissimilarity. ns, not significant; * significant at *p* < 0.05; ** significant at *p* < 0.01; *** significant at *p* < 0.001.

High-throughput 16S rRNA gene amplicon sequencing was performed to characterize changes in soil bacterial community composition and diversity under different amendment treatments. We first assessed bacterial α-diversity across the treatments. The GM40% treatment harbored the highest number of observed ASVs (Figure 8b), and the Chao1 index, reflecting estimated bacterial richness, was also relatively high under GM40% (Figure 8c). We then examined the relative abundance of bacterial taxa at the phylum level. Pseudomonadota, Actinomycetota, and Bacteroidota were among the dominant bacterial phyla across the treatments (Figure 8a). The relative proportions of these dominant phyla differed between the M group and GM group, with marked variations in the relative abundances of Pseudomonadota, Actinomycetota, and Bacteroidota across the different application rates. Principal coordinate analysis (PCoA) further revealed distinct distribution patterns of bacterial communities among the treatments (Figure 8d). PC1 and PC2 explained 47.2% and 15.0% of the total variation, respectively, together accounting for 62.2% of the variation in bacterial community composition. The CK, M and GM groups exhibited distinct distributions in the ordination space, with GM group samples generally separated from the CK and M group samples along PC1. The M group was primarily distributed on the negative side of PC1, whereas the GM group was mainly located on the positive side.

## 4. Discussion

Increasing evidence has highlighted the considerable potential of coal gangue, an abundant solid waste, for the remediation of saline–alkali soils [17]. However, the relatively limited interfacial properties of raw coal gangue may constrain its interactions with the soil environment and consequently limit its amendment efficiency. In the present study, organic modification substantially altered the surface properties of coal gangue and was accompanied by enhanced soil improvement and soybean growth under saline–alkali conditions.

Characterization of the two materials, M and GM, indicates that AEO-7 altered the surface chemistry of the gangue without changing its mineral framework. The FTIR bands of kaolinite and quartz were retained after modification, whereas the new absorption at 2800–3000 cm^−1^ indicates an adsorbed alkyl layer [27]. The contact angle decreased from 57.2° to 16.4°, that is, the surface became markedly more water-wettable. Ethoxylated surfactants can also hydrophobize mineral surfaces, depending on whether the alkyl tails or the EO chains are exposed at the interface [28]. The decrease observed here indicates that the EO segments remained exposed to the aqueous phase, plausibly because the alkyl tails are associated with the carbonaceous fraction of the gangue. The simultaneous decrease in specific surface area and increase in mean pore diameter is the signature of pore blocking by the adsorbed layer rather than of a change in mineral texture [29].

Consistent with these changes in interfacial properties, GM group application significantly increased soil alkali-hydrolyzable N, available K, and soil organic matter; decreased soil pH; and alleviated the decline in available P caused by coal gangue application (Figure 2). The increase in available K under GM may partly originate from the release of K from coal gangue, while the altered wettability following modification may further promote K release into the soil solution [30]. In contrast, the decline in available P may be associated with the interaction of phosphate with soil Ca and subsequent P fixation [31]; the smaller decline under the GM group may indicate that organic modification altered the contact between coal gangue and soil and, consequently, the P fixation process [32]. Previous studies have shown that sulfur-containing minerals in coal gangue can release H^+^ during leaching, thereby promoting the neutralization of alkaline soils [33]. In addition, the organic components introduced by AEO-7 and the microaggregates formed by the modified amendment may favor organic carbon stabilization and physical protection, thereby promoting the accumulation of soil organic matter [34,35]. The improvement in soil nutrient and organic matter status was further accompanied by enhanced soil enzyme activities [36]. Meanwhile, the increases in pore volume and mean pore diameter of the GM group may alter water and gas transport within the soil system [37], providing a more favorable microenvironment for microbial activity and organic matter transformation [16].

To further validate the practical effectiveness of modified coal gangue in improving saline–alkali soil, a soybean pot experiment was conducted to evaluate its effects on plant growth and physiological characteristics. In the present study, GM group significantly increased soybean biomass, plant height, and root length compared with the M group (Figure 5a–j), indicating that organic modification further enhanced the capacity of coal gangue to promote plant growth [38]. In addition to biomass responses, the GM group also markedly improved soybean physiological performance. Saline–alkali stress can inhibit plant growth by disrupting photosynthetic processes and inducing reactive oxygen species accumulation [39], whereas the GM group in the present study significantly increased the net photosynthetic rate and transpiration rate of soybean, accompanied by increased SPAD values (Figure 5l), indicating improved leaf photosynthetic performance under the GM group [40]. Meanwhile, the enhanced activities of POD, SOD, and CAT indicated a positive change in the antioxidant defense capacity of soybean [38]. The coordinated improvement in photosynthetic performance and antioxidant enzyme activities may help maintain normal physiological metabolism and carbon assimilation, thereby promoting dry matter accumulation and plant growth [41]. Overall, GM application was accompanied by improvements in the saline–alkali soil environment, enhanced plant antioxidant defense, and improved photosynthetic performance, ultimately resulting in increased soybean growth and biomass accumulation [42].

Soil microbial communities play important roles in nutrient cycling and the maintenance of overall soil functioning, and changes in soil nutrient concentrations are closely associated with microbial community structure [43]. Previous studies have shown that coal gangue can alter soil properties and consequently affect bacterial and fungal community structures [44]. However, the effects of raw coal gangue alone on microbial community structure in saline–alkali soils may be relatively limited, possibly due to its physicochemical and interfacial properties. In the present study, GM increased the number of soil bacterial ASVs, particularly under GM40% (Figure 8b); alpha diversity analysis showed that the Chao1 richness index was significantly higher in the GM20% and GM40% treatments compared to the M groups (Figure 8c); beta diversity analysis using Bray-Curtis PCoA clearly separated the CK, M, and GM groups into distinct clusters (Figure 8d), indicating that the modification significantly shifted the overall bacterial community composition and significantly altered the bacterial community structure of saline–alkali soil, with increased relative abundances of Actinomycetota and Bacteroidota (Figure 8a) [45]. Bacteroidota are generally considered nutrient-responsive taxa that preferentially grow in soils with greater nutrient availability [46], and their increased abundance may be associated with improvements in soil nutrient and organic matter status following GM application [44]. Similarly, Actinomycetota are known to play crucial roles in the decomposition of complex organic matter and the suppression of soil-borne pathogens, which aligns with the observed increase in soil enzyme activities. These results collectively demonstrate that the enhanced interfacial wettability and nutrient delivery of GM significantly reshaped the soil microbial community towards a more metabolically active state.

## 5. Conclusions

In this study, organically GM was fabricated and adopted as an amendment for saline–alkali soils. Experimental characterizations reveal that, compared with M, GM contains higher carbon and sulfur contents and exhibits distinctly aggregated microstructural morphology. Both M and GM can modulate physicochemical properties and microbial community abundance of saline–alkali soils, thereby regulating nutrient uptake and physiological metabolism of crops to further mediate biomass accumulation. Nevertheless, M delivers far inferior comprehensive remediation performance relative to GM. Pot trials with soybean further validate that GM markedly improves multiple crop physiological parameters.

These findings highlight the great potential of surfactant-modified solid waste as a high-performance composite amendment for saline–alkali soil remediation. However, this study serves as a mechanistic and short-term screening rather than a direct field recommendation. Because the modifier also contained molybdenum and magnesium, their synergistic nutritional effects could not be completely isolated from the surfactant in this design. Additionally, long-term environmental safety requires further validation. Future research will build on these mechanistic insights to conduct multi-season field trials at agronomically feasible rates, further optimizing this waste-to-resource strategy for sustainable agriculture.

## Figures and Tables

**Table 1:** Content of the top five elements in the experimental coal gangue (mass%).

Tested Materials	C	Si	Al	S	Fe
M	19.4885	6.0963	5.5169	1.2019	0.7594
GM	20.3671	4.8675	4.4935	1.4169	0.7575

M: raw coal gangue; GM: modified coal gangue.

**Table 2:** Concentrations of the five major oxides in the experimental coal gangue (mass%).

Tested Materials	CO_2_	SiO_2_	Al_2_O_3_	SO_3_	Fe_2_O_3_
M	71.4075	13.0419	10.424	3.0011	1.0857
GM	74.6269	10.4131	8.4903	3.5379	1.0831

M: raw coal gangue; GM: modified coal gangue.

**Table 3:** Trace element contents in coal gangue and screening values for agricultural land pollution risk.

Pollutant	Trace Element Contents in Group M/(mg/kg)	Trace Element Contents in Group GM/(mg/kg)	Screening Values for Agricultural Land Pollution Risk (pH > 7.5) /(mg/kg)
As	22	16	25
Pb	12	8	170
Cd	0.3	0.2	0.6
Cr	84	59	250
Cu	35	27	100
Ni	33	30	190

M: raw coal gangue; GM: modified coal gangue.

**Table 4:** Pore-structure parameters of coal gangue before and after modification.

Tested Material	Specific Surface Area (m^2^/g)	Pore Volume (cm^3^/g)	Mean Pore Size (nm)
M	2.2674	0.0065	11.4669
GM	1.6626	0.0077	18.5252

M: raw coal gangue; GM: modified coal gangue.

## Data Availability

The data presented in this study are available upon request from the corresponding author due to confidentiality constraints.
